# The Emerging Roles of the Cephalic Neural Crest in Brain Development and Developmental Encephalopathies

**DOI:** 10.3390/ijms24129844

**Published:** 2023-06-07

**Authors:** Emmanuel Bruet, Diego Amarante-Silva, Tatiana Gorojankina, Sophie Creuzet

**Affiliations:** Paris-Saclay Institute of Neuroscience, NeuroPSI, CNRS, Paris-Saclay University, Campus CEA Saclay, Bât 151, 151 Route de la Rotonde, 91400 Saclay, France

**Keywords:** neural crest, mesectoderm, embryology, evolution, forebrain development

## Abstract

The neural crest, a unique cell population originating from the primitive neural field, has a multi-systemic and structural contribution to vertebrate development. At the cephalic level, the neural crest generates most of the skeletal tissues encasing the developing forebrain and provides the prosencephalon with functional vasculature and meninges. Over the last decade, we have demonstrated that the cephalic neural crest (CNC) exerts an autonomous and prominent control on the development of the forebrain and sense organs. The present paper reviews the primary mechanisms by which CNC can orchestrate vertebrate encephalization. Demonstrating the role of the CNC as an exogenous source of patterning for the forebrain provides a novel conceptual framework with profound implications for understanding neurodevelopment. From a biomedical standpoint, these data suggest that the spectrum of neurocristopathies is broader than expected and that some neurological disorders may stem from CNC dysfunctions.

## 1. Introduction

The face and the brain are phylogenetic organs that formed during vertebrate evolution. Their development coincided with the emergence of a multipotent cell population in the vertebrate embryo, the neural crest (NC). This structure represents an essential asset in the evolution of the chordate phylum since specific vertebrate traits (peripheral nervous system, cephalic skeletal tissues, and head development) are linked to the NC and its derivatives [1,2]. The NC first develops from the neural fold, bulging from the lateral borders of the neural plate at the neurula stage. At the time of neural tube closure, NC cells delaminate and migrate from the neural primordium to colonize elected sites, where they differentiate into a large variety of cell types. NC derivatives include the neurons and glial cells of the peripheral nervous system, the pigment cells, and endocrine cells, such as the adrenal medulla and calcitonin-producing cells [2].

Anteriorly, the cephalic NC (CNC) forms the mesectoderm, a vast, multipotent, and plastic mesenchyme which differentiates into connective tissues, chondrogenic and osteogenic cells. Consequently, CNC cells form much of the craniofacial skeleton, including the skull, the upper and lower jaws, and the hypobranchial skeleton [3]. In addition to skeletal derivatives, the CNC also contributes to the elaboration of the facial and cerebral vascular tree: CNC cells give rise to the pericytes lining the facial and cerebral capillaries in the forebrain and the adventitial peri-vascular cells of the aortic arches. Its contribution to the functional vasculature in the anterior part of the body extends down to the heart, where the CNC cells form the conotruncus and the sigmoid valves [4,5]. Therefore, the cardio-vascular CNC derivatives participate in the homeostasis of the craniofacial structures and support the requisite demands in the oxygenation of the developing forebrain. Thus, from a developmental standpoint, the CNC exerts an essential structural role in building skeletal, vascular, and cephalic structures.

As an echo of the multiple and diverse contributions of NC cells, the spectrum of NC dysfunctions is broad and encompass a multiplicity of pathogenic conditions. The cohort of disorders (like melanoma, congenital melanocytic naevus, Hirschsprung disease, neurofibromatosis, and Merkel cell carcinoma) and malformative syndromes (Waardenburg, CHARGE, DiGeorge, Goldenhar, Axenfeld–Rieger, cranio-frontonasal syndromes, and congenital heart defects) arising from NC defects are collectively referred to as neurocristopathies [6,7]. The congenital disorders and craniofacial dysmorphisms that stem from NC defects illustrate how the basic embryological mechanisms and aberrant variations in molecular network activity may diverge from the normal developmental program. The conservation of cell interactions and signaling pathways across species justifies the importance of experimental animal studies to enlighten the etiology of human diseases. Over the last decade, investigations using developmental models have shown that the CNC, besides its structural role in head development, also exerts a potent morphogenetic “paracrine” effect on the brain and sense organs. Here, we review the primary pathway by which CNC cells control vertebrate encephalization. The evidence indicate that the spectrum of neural crest disorders is broader than expected and suggest that some neurodevelopmental defects and encephalopathies are neurocristopathies.

## 2. Vertebrate Neurulation: Ontogenesis of the Central Nervous System and the Neural Crest

**Figure 1:**
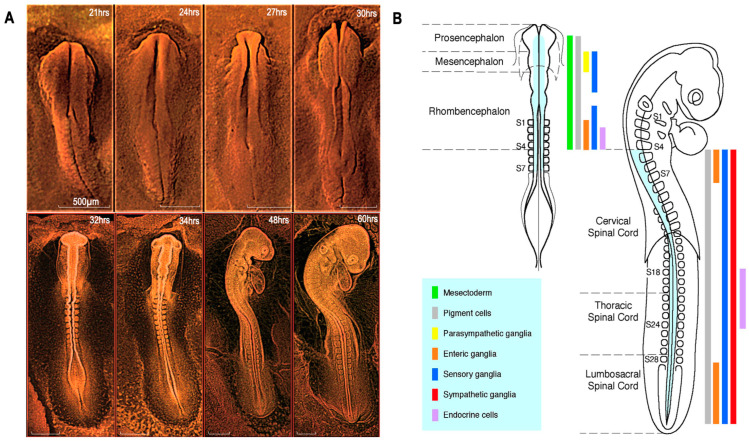
Development of the nervous system in the bird embryo. (**A**) Embryos showing the dynamics of early and then late stages of neurulation. From left to right, the cephalic development of chicken embryos after 21 h, 24 h, 27 h (showing the closure of the mesencephalic neural tube), 32 h (showing the first constrictions individualizing the anterior, middle, and posterior brain), 34 h (showing the segmentation of the rhombencephalon into rhombomeres is apparent), 48 h of incubation (showing the subdivision of the prosencephalon into the telencephalon and diencephalon) and 60 h. (**B**) Derivatives of the NC according to their level of origin along the anteroposterior axis. At the cephalic level, the NC forms mesectodermal derivatives (green) comprising skeletogenic, connective, adipose and perivascular cells. Glial and melanocytic phenotypes represent a basic trait produced throughout the neuraxis. The rhombencephalic and sacral NC provides the enteric neurons. As for the neurendocrine, calcitonin and adrenomedullary cells, they derive from the posterior rhombencephalic (r8) and thoracic levels, respectively. Scale bars: 500 µm. Adapted from [13].

The formation of the nervous system in vertebrate embryos begins at the late stage of gastrulation with a thickening of the superficial sheet. It leads to the formation of a neural plate, a specification of the dorsal ectoderm, which, under the induction of the underlying axial mesoderm and the notochord/prechordal plate complex, thickens along the midline and delimits the future neural ectoderm or neurectoderm, and initiates the formation of the central nervous system (CNS; Figure 1A).

At this stage, a neural fold bilaterally flanks the neural plate and accompanies the growth, elevation, and folding of the neural plate towards the midline. During this process, the neurectoderm separates from the superficial ectoderm, destined to form the skin. As the edges of the plate curve and form a neural groove, the neural bulges tend to converge along the dorsal midline. This complex growth-convergence process results in the fusion of the lateral edges of the neurectoderm and leads to neural tube closure [8,9,10].

Neural tube formation starts at the cephalic level before progressing to the more caudal levels. In neurula-stage embryos, while the anterior part of the embryo sees the progressive formation of the CNS, in the posterior part, gastrulation is still very active at the primitive streak. However, this folding and closing dynamic, characteristic of primary neurulation, only concerns the anterior 2/3 of the neuraxis. More caudally, secondary neurulation occurs due to the cavitation of a neural cord [11,12].

Anteriorly, soon after neural tube closure, the organization of the encephalon into three vesicles is fixed: the anterior brain or prosencephalon, the midbrain or mesencephalon, and the posterior brain or rhombencephalon (Figure 1A). More caudally, the neural tube is the origin of the spinal cord. Later on, the prosencephalon subdivides secondarily into the telencephalon and diencephalon. The telencephalon gives rise to the cerebral hemispheres, the olfactory bulbs, and the hippocampus. From the diencephalon comes the thalamus, the epithalamus, and the hypothalamus.

Parallel to the formation of the CNS, neurulation also leads to the formation of a population of pluripotent cells, the NC, which appears at the edge of the neural plate as bilateral neural bulges interposed between the neural and superficial ectoderm. As the two neural bulges fuse to form the neural tube, NC cells undergo an epithelial–mesenchymal transition: they detach from the neural plate and become mesenchymal. At the same time, NC cells acquire migratory properties and disperse throughout the embryo to give various derivatives, such as neurons, glial, pigmentary, and endocrine cells, but also cartilaginous and bony skeletal tissue [2]. Due to its neural and glial derivatives, NC is essential for forming the enteric nervous system (ENS) and developing the peripheral nervous system (PNS).

## 3. Neural Crest Discovery and Ontogenic Contribution

The NC “emerged” only recently in the history of biology. A German anatomist, His W., discovered NC cells originating from the edges of the neural tube in the chicken embryo, which migrated to form the spinal ganglia [14] and coined the term “ganglion crest”. A few years later, Kastschenko N., who described the mesenchymal contribution of NC cells to branchial arches in selachian embryos, first reported the role of NC in the formation of facial cartilages and branchial arches, and Goronowitsch N. extended this notion to birds and teleost fish [15,16]. Notably, Platt J.B., an American researcher who, based on her observations in the newt, *Necturus*, claimed that the NC was at the origin of the “visceral” (i.e., gill) cartilages and assigned the origin of dentin to the NC [17,18]. Moreover, she ascribed the term “mesectoderm” or “ectomesenchyme” to the NC-derived mesenchyme in order to distinguish it from the mesoderm-derived mesenchyme. At that time, the “three germ layers” theory prevailed, which assigned an exclusive mesodermal origin to skeletal tissue in vertebrates; these observations were particularly disputed. The experimental demonstration came later, during the first half of the 20th century, by experiments essentially based on ablation, xenograft, and cell tracing experiments carried out in lower vertebrates, amphibians, and fish [19,20,21,22,23,24,25,26]. During the second half of the 20th century, these notions were extended to higher vertebrates, birds, and mammals. At the beginning of the 1960s, the work on the chicken embryo regained interest and intensity, such as applying tritiated thymidine to elective embryonic territories which allowed a precise cell marking [27]. This approach allowed to trace the dissemination of the NC cells from the neural primordium to the facial processes and branchial arches [28]. At the end of this decade, the quail–chick chimera technique made a breakthrough in experimental embryology [29]. It used interphase heterochromatin distribution as a differential structural character between two bird species. This character enables the discrimination of discrete cell territory to follow the fate of its progenies in an interspecific environment. While in chicken heterochromatin evenly disperses in the nucleoplasm, in quail, it condenses to the nucleolus; this structural distinction allowed tracing the respective contribution of tissues from each species, which later on, benefited from developing an antibody (Ab) directed against an antigen carried by quail cell nuclei, QCPN Ab (Carlson & Carlson, 1984; University of Michigan), to ease the histological analysis of chimeric tissues. Because of its stability and resolution, irrespective of the cell state of differentiation, this technique became an important tool well-adapted to the systematic exploration of NC cells’ fate (for review [3,30]). By combining the micromanipulation of tissue Anlagen and gene expression, this technique becomes a resourceful model for functional studies. This model offers the prospect of unraveling cell interactions to identify the mechanisms underlying cell specification and differentiation with unparalleled spatiotemporal accuracy. It became decisive for the systematic explorations of the NC to understand the complexity of the NC cell population, to decipher the bidirectional interactions with the adjacent tissues, and more specifically to understand the developmental crosstalk with the developing brain [31].

## 4. CNC: A Source of Mesenchyme for the Face and Pharynx

The NC has the dual characteristics of being transient and multipotent. Although it disappears as an anatomical structure, undergoing an epithelial–mesenchymal transition, it produces highly invasive cells that have a lasting impact on vertebrate ontogeny through their derivatives. The NC has a multisystemic contribution. On one hand, it is at the origin of various derivatives, such as neurons of the peripheral nervous system, glial, pigmentary, and endocrine cells. However, the nature of the derivatives it generates varies at the level of the anteroposterior axis from which its cells originate (Figure 1B).

**Figure 2:**
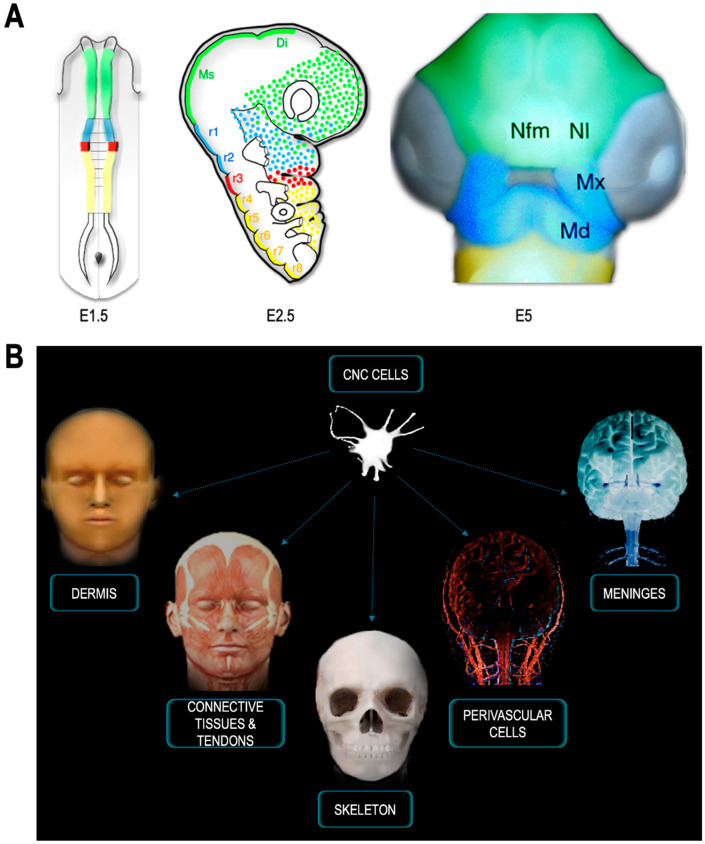
Contribution of the CNC to head formation and craniofacial structures. (**A**) Diencephalic and mesencephalic CNC cells colonize the medial nasofrontal (Nfm) and nasolateral (Nl) buds. The anterior rhombencephalic NC (blue) provides most of the mesenchyme for the maxillary (Mx) and mandibular (Md) processes. (**B**) CNC cells differentiate into the dermis, the connective tissue, and tendons of the extraocular, facial and gill striated muscles, the skull and jaw skeleton. The CNC is also the origin of the perivascular cells that accompany the vascular network for vascularization of the face and forebrain, as well as the meninges of the telencephalon and thalamus. Adapted from [48].

In birds, the migration of the CNC begins in the midbrain, when the neural bulges meet at the dorsal midline. Before resorbing into a population of migratory and mesenchymal cells, the NC cells form two easily identifiable bilateral masses. Their complete emigration at the cephalic level covers about 10 h (from 6 to 13 somite stage). In the early stages of their migration, the cells express genes that allow them to be distinguished from the neural primordium, from which they originate, and from the surface ectoderm. These include transcription factors, such as *Slug*/*Snail2*; genes of the *Sry-related HMG box* (*Sox*) family, notably *Sox8*, *Sox9* and *Sox10*; *Paired box* (*Pax*)*3*; *Pax7*; and *Forkhead box* (*Fox*)*d3* [32,33,34,35,36,37]. During this period, they also acquire surface antigens and, in particular, a glycoprotein recognized by a monoclonal antibody, Human natural killer-1 (HNK1) is widely used to follow the initial stages of their migration [38]. However, none of these genes used as markers can completely characterize the whole population of NC cells; moreover, their expression is usually transient. The chimera system provided a reliable approach for short- and long-term tracing. According to quail–chick fate maps, NC cells from the posterior diencephalon (located caudally to the pineal gland anlage) colonize the upper face primordium, corresponding to the nasofrontal and nasolateral buds (Figure 2A). The NC cells from the mesencephalon and the first two rhombomeres r1–r2, fill the first branchial arch (BA1; Figure 2A). In the more posterior branchial arches (BA2-BA4-6), cells originate from the middle and posterior rhombencephalon. NC cells from r4 provide the mesectodermal cells in BA2, with a small contribution from adjacent rhombomeres r3 and r5. NC cells from r6 to r8 colonize BA3 and BA4-6. These notions were primarily observed in birds but were extended to mammals via the development of specific transgenic models [39,40,41,42,43,44,45,46,47].

The migration of the NC cells primarily depends on the extracellular matrix where fibronectin, collagens, and laminins, delimit permissive migration pathways [49,50,51]. Repellent signals are produced along the migration pathways, whereas chemoattractant molecules are present within the migration pathways and at their destination site. In particular, the interaction between the molecule Semaphorin 3A and its receptor Neuropilin2 exerts a potent role in segregating migration currents from the rhombencephalic NC to the posterior arches [52,53]. However, Semaphorin acts in conjunction with other molecules to define “crest-free zones”, where NC cells are absent. The combination of permissive versus repellent signals confers a segmental distribution to the migration of these highly invasive and pluripotent cells. When the genes encoding the transcription factor *Twist*, or the neuregulin receptor, *ErbB4*, are mutated, an abnormal and invasive migratory phenotype of crest-free zones occurs [54,55,56].

Similarly, when the binding of Ephrin molecules to their receptors is inhibited, directional migration of NC cells is impaired with long-term consequences of craniofacial malformations [57]. In addition, local foci of cell death also refine the migration of NC cells derived from r3 and r5, which undergo massive apoptosis under the control of adjacent rhombomeres through the production of the factor Bone Morphogenetic Protein 4 (Bmp4). This results in a depletion of mesectodermal cells at the interface between the 1st and 2nd BAs and the 2nd and 3rd BAs, which restricts r4 cell migration to BA2, whose mesenchymal contribution is thus isolated from the adjacent arches [58,59,60]. Together, these mechanisms contribute to the restriction of a cell population in the second arch with a robust molecular identity inherent to its exclusive expression of *Homeobox* (*Hox*)*a2* gene, whose activity is potentially deleterious for the differentiation of more rostral structures. Furthermore, some molecules can guide CNC cell migration. They belong to the families of stromal cell-derived factors (Sdf), vascular endothelium growth factors (Vegf), Fibroblasts growth factors (Fgf), and Glial cell-derived neurotrophic factor (Gdnf) (for review [61]). Most of these guiding molecules exert a dual role: on one hand, they pave the way for NC cell migration, and on the other hand, they control NC cell differentiation. Their dual action provides a functional link between the multiple morphogenetic solicitations at the early stages of NC cell migration and the multisystemic contribution of NC derivatives to morphogenesis.

## 5. The CNC and Craniofacial Skeletogenesis: A Vertebrate Synapomorphy

The craniofacial skeleton represents a complex system of interconnected skeletal pieces presenting tremendous morphological variety. Moreover, the craniofacial skeletal system is also composite, consisting of various tissues, cartilage, endochondral bone, and membrane bone. In addition to the anatomical and histological complexity of its components which is challenging to study, this system develops according to a global organization plan which fulfills multiple functions and requirements. They include the protection of the brain, the support of the sense organs, the formation of the organs of predation, and the anchoring point of the digestive and superior respiratory tracts. From these varied needs, distinct skeletogenic processes are engaged: in the upper face that includes, on one hand, the elaboration of the cranium and the organization of the sensory capsules, which collectively form the neurocranium, and, on the other hand, includes the construction of the jaws and the hypobranchial skeleton, which together compose the visceral skeleton or viscerocranium, also referred to as splanchnocranium. These ontogenic processes follow their intrinsic morphogenetic logic, resulting from distinct developmental strategies initiated from isolated skeletogenic foci. As their differentiation progresses, these different skeletogenic foci become confluent and inter-dependent. Despite their apparent complexity, craniofacial structures share the common feature of being derived from the CNC. Cell tracing investigations in the chicken [28,62,63,64,65,66] revealed that most of the cranial skeleton is NC-derived. They are at the origin of the bones and cartilage of the upper and lower jaws, the nasal capsule, and the periorbital skeleton [63,66], along with the visceral, maxillo-mandibular, and hyoid skeleton [67,68]. In addition, in mammals, NC cells are also at the origin of the dentin, cementum, and dental pulp [39]. In contrast, only the occipital region, the basi-post-sphenoid, and part of the otic capsule are of mesodermal origin. The contribution of mesoderm to the cephalic structures of vertebrates is also to form the vascular endothelium of the blood vessels, necessary for nutrition and oxygenation and the striated muscles of the face. Dorsally, the boundary between mesectodermal and mesodermal components of the cranial skeleton lies at the interface between the parietal and occipital bone. Ventrally, the boundary coincides with the anterior end of the notochord, which projects to the level of the sella turcica housing the ventral face of the pituitary gland, where the basi-post-sphenoid of mesodermal origin abuts to the basi-pre-sphenoid of NC origin.

## 6. Functional Partition in CNC Skeleton and Molecular Identities

Long-term tracing studies revealed different capacities in CNC cells to form skeletal tissues along the neuraxis. The ability to form cartilage and endochondral bone is shared by all CNC cells, regardless of their level of origin. However, the ability to form dermal bone is limited to the CNC cells present in the face, the nasofrontal and lateral buds, and the maxillomandibular processes. The CNC can therefore be subdivided into two functional areas. One area is rostral, extending from the middle diencephalon down to r2, which is the origin of the facial and cranial skeleton. Therefore, this region of the CNC is considered as the “facial NC” (or FNC [69]; Figure 2A). The other is more caudal, from r4 to r8, which allows the cells to form much of the hyoid bone. At the interface between these two territories, the cells of r3 correspond to an intermediate zone where NC cells participate in the first arch, facial, and the second arch, hyoid [69]. The functional demarcation within the CNC correlates with distinct molecular identities: anteriorly, the CNC cells originating from the diencephalon down to r2 do not express *Hox* genes, while CNC cells from r4 down to r8 express a sophisticated code of *Hox* genes. During development, the activation of Hox genes governs tissue segmentation and the antero-posterior patterning of the CNS. The expression of *Hox* genes is also intimately linked to the specification of the rhombencephalic NCC cells destined to fill the pharyngeal arches, and restrict the skeletogenic capacities to endochondral cartilage and bone derivatives in the gill region [70,71,72]. Anteriorly, in the frontonasal and maxillomandibular region, *Hox* genes are not expressed.

Ectopic transpositions of CNC demonstrated that within the *Hox*-negative domain, the cells of the facial NC behave as an “equivalence group”, i.e., a morphogenetic territory whose different levels are endowed with comparable developmental capacities. Each portion of the FNC is equally capable of generating the whole facial and mandibular skeletal components. In contrast, the transposition of the *Hox*-positive cells into the *Hox*-negative domain could not modify their Hox code. These cells migrate and colonize the facial buds, but are unable to form any appropriate skeletal, nasofrontal and maxillomandibular structures [70,73]. In mice, null mutation of the *Hox* genes perturb the formation of skeletal structures derived from the CNC [71,72,74], and results in duplication of the lower jaw, at the expense of BA2 skeleton [71,72]. Similarly, heterotopic grafts or ectopic expression of *Hoxa2* demonstrate the incompatibility between *Hox* gene expression and the development of maxillo-mandibular structures: Hox-positive cells in BA1 cannot generate a mandibular skeleton [69,73,75,76,77].

## 7. Contribution of CNC to Cardio-Vascular and Peri-Ocular Structures

In addition to the head skeleton, CNC massively contributes to connective tissues and tendons of the facial striated musculature (Figure 2A,B). The CNC forms the smooth musculature of the arrector pili muscles and the subcutaneous adipose tissue [62]: it provides the attachment points and connective sheaths for the muscle bundles, and dictates the arrangement of the hyoid, maxillomandibular, and extraocular skeletal musculature [45,67].

The CNC cells also contribute to the singularity of the anterior vascular arborescence: they form the perivascular cells which line the arterial tree from its emergence in the conotruncus to the capillary network irrigating the face and the forebrain. CNC cells migrating from r6 to r8, build the interventricular septum and the sigmoid valves [34,40,78,79,80,81]. In addition, they participate in the vasa vasorum in the ascending aorta and the tunica media of the coronary arteries [82]. When these cells are absent or deficient, irreversible defects affect the septation of the heart and the systolic ejection chambers [78,79,81,83]. These defects stem from the aberrant proliferation of cardiomyocytes and compromise the formation of myofibers. Secondarily, such defects are complicated by extensive coronary atresia of the entire myocardium [84,85,86,87,88]. Given the vital importance of the CNC in cardiac development and physiology, the territory extending from r6 to r8 is classically considered as the “cardiac NC”.

**Figure 3:**
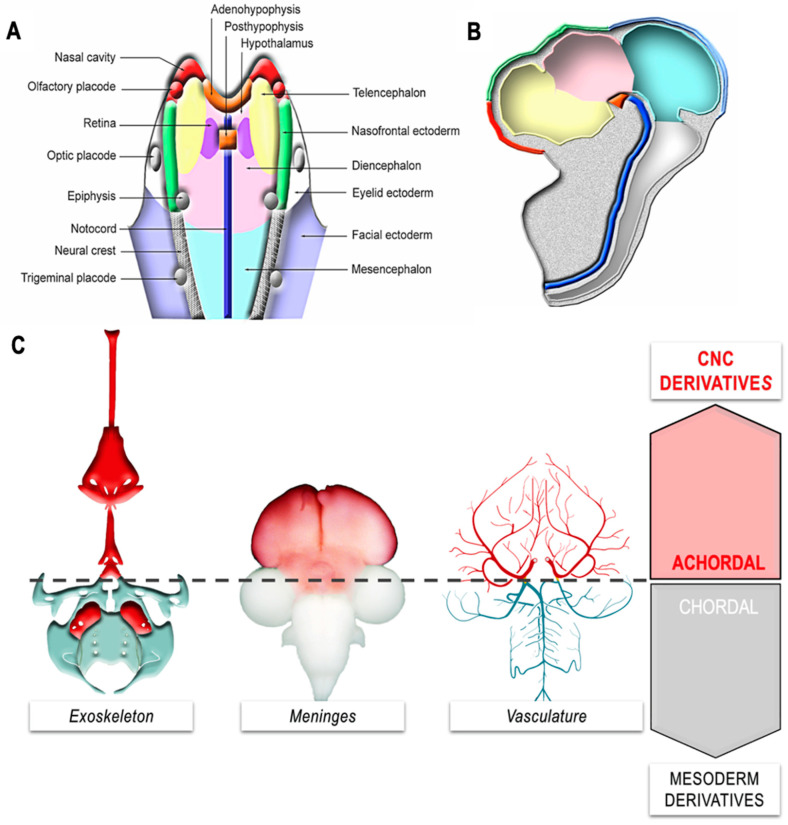
Development of the achordal segment (**A**) Projection of presumptive territories at the neural plate stage, showing the outline of the pituitary (orange), nasal muscularis (red), nasofrontal ectoderm (green) and telencephalon (yellow). In projection, the notochord appears in blue. (**B**) Fate of neuroectodermal derivatives at E5. (**C**) Derivatives of the CNC and emergence of the achordal segment. The skeletal, meningeal, and perivascular derivatives of the CNC that accompany the growth of the anterior cerebrum develop anterior to the tip of the notochord. Adapted from [89].

In the face and the brain, the FNC has a musculo-connective contribution to the vasculature: its cells differentiate into pericytes and can be readily distinguished from endothelial cells by the accumulation of smooth muscle actin filament in their cytoplasm [4,5]. In the forebrain, pericytes derived from the CNC cells line the capillary network forming a very dense choroidal veil that prefigures the formation of the leptomeninges [4,66]. The meninges of the telencephalic hemispheres and thalamus thus originate from the CNC (Figure 2B). More caudally, from the optic tectum down to the tip of the spinal cord, meninges form the mesoderm. The transition between the vascular sectors, lined by CNC and mesoderm, occurs in the Circle of Willis, around the pituitary gland (Figure 3).

Laterally, the CNC also produces a tissue similar to the pia mater around the ocular anlage, the choroid membrane, which lines the outer surface of the retina, thus organizing the posterior uvea. In the choroid membrane, CNC cells differentiate into pericytes and melanocytes [4,64,66,86]. The CNC phenotype consists of perivascular cells in the periocular mesectodermal layer intimately associated with the pigmented retina. It also forms the cartilaginous skeleton of the sclera, the membranous bones that form the scleral ossicles. In the anterior periocular region, the abundant mesenchymal tissue derived from CNC also yields smooth muscle cells in the ciliary bodies and around Schlemm’s canal, as well as the striated muscle myofibers of the iris (for review [86]). The contiguous relationship between these smooth and striated muscles derived from the CNC extends to the stroma of the nictitating membrane and the eyelids [86]. Lining the anterior chamber, the CNC form the corneal endothelium and keratocytes of the corneal stroma [64], which in many respects is suggestive of a cartilaginous-like structure. At the interface between the sclera and the cornea, CNC cells persist in an undifferentiated state in adults and form a stem cell niche within the scleral-corneal lamina. Altogether, the periocular CNC derivatives contribute to visual function by providing ocular structures with refraction media (cornea, and ossicles) and by participating in their homeostasis, both at the level of the anterior chamber (ciliary body and muscles, Schlemm’s canal), and the posterior chamber (choroidal membrane; for review [86]).

## 8. CNC Contribution to the Emergence of an Achordal Segment: The “New Head”

During evolution, the transition from protochordates to vertebrates was marked by the appearance of a new structure, the NC. Absent in protochordates, the NC is a vertebrate innovation, a synapomorphic character unique to this group: given the diversity of lineages it generates, the NC can be considered as a fourth germ layer, making vertebrates quadroblastic organisms [90,91,92].

Anterior to the pineal anlage, the neural fold does not produce migrating CNC [90,93,94], but gives rise to the pituitary anlage medially (Figure 3A–C), which is contiguous with the rostral end of the notochord in early neurula. From the mesio-lateral territories of the neural fold, the nasal mucosa, the olfactory placodes, as well as the ectoderm destined to cover the naso-frontal bud and the phyltrum region develop. In contrast, at this stage, the telencephalon is located more caudally in the cephalic neural plate, laterally flanking the presumptive territory of the retina and the diencephalon (Figure 3A,B). It is important to note that all of the skeletal, perivascular, and meningeal derivatives of the facial CNC that accompany the development and growth of the forebrain develop anteriorly to the rostral end of the notochord (Figure 3A,B), whose anatomical projection onto the CNS corresponds to the level of the hypophysis.

From a phylogenetic standpoint, the CNC cells have allowed the emergence of a new territory, the achordal segment characterized by innovative facial and cerebral structures (Figure 3C). The transition from cephalochordates to vertebrates was marked by the sophistication of the brain and its associated sense organs—vision, smell, hearing—along with the development of facial structures and predatory organs. While cephalochordate fed by filtering organic marine particles, the vertebrates became predators. These transformations were accompanied by a change in lifestyle, a change in behavior that involved the acquisition of predatory organs, and the sophistication of sense organs and associative nerve centers. This change coincided with a considerable increase in the volume and complexity of cephalic vesicles in vertebrates, associated with the development of cognitive functions.

The importance of CNC in the construction of the vertebrate head, revealed in experimental embryology studies, paved the way for the hypothesis presented by Gans and Northcutt in a 1983 paper entitled “Neural Crest and the Origin of Vertebrates: A New Head” [1]. The authors argue that the appearance of the CNC in the embryo of the first vertebrates was a decisive step, playing a key role in the formation of a “new head”, an innovative addition to the pre-existing chordate organizational plan. This “new head” was the site of the development of a brain associated with sensory organs (absent in protochordates) and a predatory organ, the jaw, whose skeleton is entirely derived from the CNC (for review [91]).

## 9. CNC Regulation of Pre-Otic Brain Morphogenesis

The hypothesis that CNC could play a prime evolutionary role in the process of cephalization has been investigated over the last years. To this end, ablation and rescue experiments were designed and extensively performed to explore the specific input of CNC cells on forebrain development.

**Figure 4:**
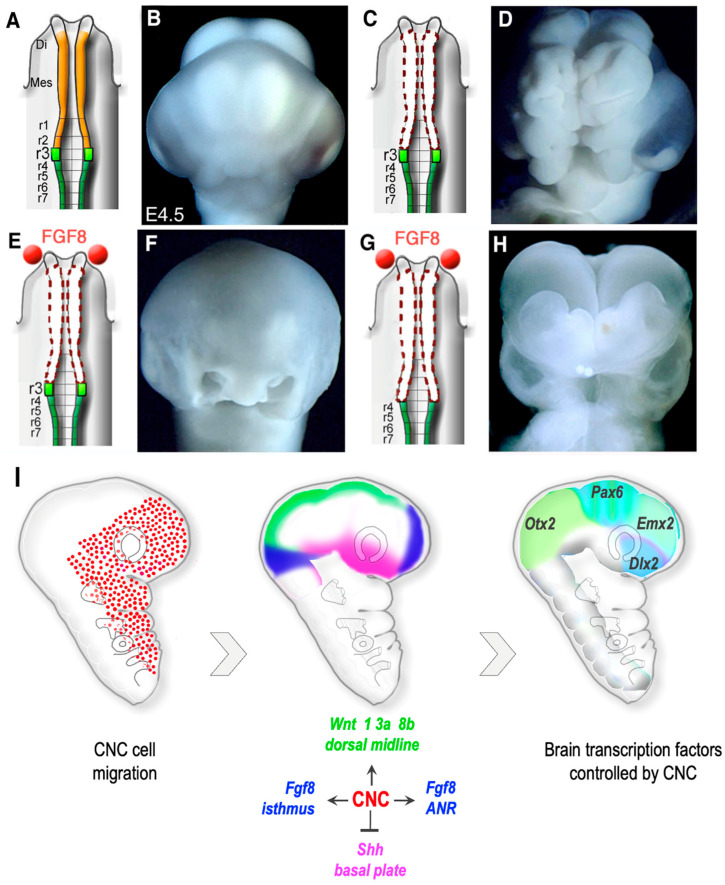
FNC controls cephalic neural tube closure. (**A**,**B**) At E5, when the facial NC ((**A**), in yellow) is intact (**B**) anterior and middle cephalic vesicle formation is normal. (**C**,**D**) Removal of the facial NC (**C**), from the middle diencephalon to r2, leads to extensive anencephaly (**D**). (**E**,**F**) Implantation of Fgf8 beads ((**E**), in red) allows closure of the tube (**F**). (**G**,**H**) When ablation is extended to r3, the embryos are anencephalic, despite Fgf8 bead implantation. (**B**) The facial NC regulates the morphogenetic activity of brain organizing centers. (**I**) During their migration, NC cells stimulate Fgf8 activity in the RNA and isthmus, as well as Wnt activity along the dorsal midline. At the same time, they limit Shh expression to the basal lamina and repress its dorsal expansion. Scale bars: 500 µm. Adapted from [99].

The absence of FNC, which results in dramatic facial phenotype, is also harmful to the brain. Microsurgical ablation of FNC led to severe anencephaly along the territories where the FNC was excised (Figure 4H). Ablated embryos showed irreversible neural tube defects, and further extended rostrally to the telencephalon [73,92,95]. Furthermore, FNC-dependent anencephaly was correlated with the loss of Fgf8 expression in the anterior neural ridge (ANR) and of the *Wingless* (*Wnt*), *Wnt1*, *Wnt3a*, and *Wnt8* genes in the dorsal part of the thalamus and the midbrain. This resulted in agenesis of the dorsal thalamic nuclei. In parallel, profound perturbations in the expression pattern of *Empty spiracles homeobox* (*Emx*)2, *Pax*6, and *Distal-less homeobox* (Dlx)2 [96,97,98] accompanied the developmental defects of cerebral hemispheres [95]. Exogenous supply of Fgf8 allows the regeneration of the development of the cerebral hemispheres (telencephalon), the thalamus (diencephalon), and the optic roof (midbrain). Fgf8 stimulates the rostral progression of CNC cells located at the limit of the excised territory (i.e., r3), which then migrate along the anterior neural plate, and promote fusion of the prosencephalic neuroepithelium (Figure 4I). At the same time, the expression of the transcription factors *Emx2*, *Pax6*, and *Dlx2*, involved in the specification of the cephalic neuroepithelium is restored [95].

Therefore, the absence of CNC generates deficits in the brain comparable to the most severe congenital malformations of the brain; these consist of partial or total absence of the cerebral hemispheres and neural tube closure defects. These observations suggest that deregulations of CNC may be at the origin of such congenital malformations, the latter considered until now as intrinsic defects of the brain tissue.

These observations suggested the existence of NC cell-dependent signaling essential for (i) neural tube closure, (ii) roof plate development, and (iii) the molecular identity of the prosencephalic lamina. By controlling the expression of Fgf8 in the ANR, the CNC exerts a “dorsalizing” action, to shape the telencephalon, and repress the ventralizing signals mediated by the *Sonic hedgehog* (Shh) morphogen (Figure 4I).

## 10. CNC Control of Brain Growth by Opposing Bmp Action

Bmps molecules are known to negatively regulate Fgf8 production in this region [99,100]. Two primary sources of Bmps exist in the anterior region of the embryo at the phylogenesis stages. One, active in the early embryo at E1.5–2, is located in the pre-chordal plate (PCP) and produces Bmp7; the other, located in the prosencephalic neuroepithelium and surface ectoderm, produces Bmp4 from E2 [101]. Factors expressed in the Spemann organizer, Chordin and Noggin, antagonists of Bmps, are involved in the control of forebrain development in the mouse [102,103]. However, CNC cells, during their migration, are a source of Bmps antagonistic factors, notably Gremlin and Noggin [104,105].

When the expression of the Bmp4 gene is driven by a retroviral vector electroporated into the ANR, its activation prevents the deployment of CNC cells in the facial processes and along the prosencephalic vesicles. This results in brain defects mainly in the absence of rhinencephalon. These deficits can be reproduced either by surgical ablation of the ANR or by locally silencing Fgf8. Depletion of Fgf8 in the ANR was accompanied by drastic reductions in upper face structures (nasal capsule and upper jaw; [106]). The significant reduction in prosencephalic size indicates that the amount of Bmp4 produced regulates the production of Fgf8 in ANR and, consequently, the volume of lateral and dorsal forebrain structures.

**Figure 5:**
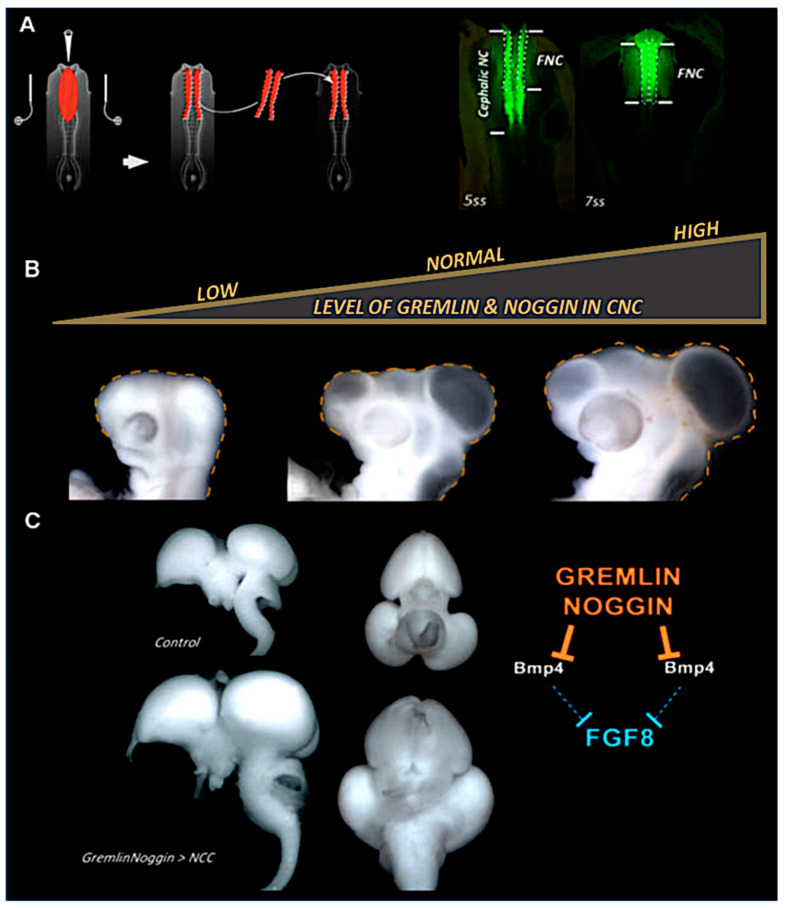
FNC regulates brain growth through the action of *Gremlin* and *Noggin*. (**A**) Bilateral transfection and grafting of the CNC. Expression of GFP to attest the transfection efficiency. (**B**) Joint inhibition of *Gremlin* and *Noggin* activity results in microcephaly (left, dotted line), whereas their overexpression results in macrocephaly (right, dotted line). (**C**) In macrocephalic embryos, brain size increases by nearly 30% more than normal. Adapted from [107,108].

Both *Gremlin* and *Noggin* are expressed in CNC cells. When these two BMP antagonists are inactivated simultaneously by bilateral electroporation with double-stranded RNAs, and then grafted onto a non-transfected host embryo, these experiments lead to a drastic reduction of Fgf8 expression in the ANR, and result in microcephaly. Conversely, when *Gremlin* and *Noggin* genes are overexpressed, *Fgf8* expression in the ANR is expanded, leading to macrocephaly with a brain size augmented by up to 30% (Figure 5; [106]).

## 11. Role of Six Genes in CNC Cells for Forebrain Development

The combined expression of the *Hox* genes constitutes a phylogenetically ancient program that underlies the organizational plan of all Bilateria. In the head, the CNC and its derivatives develop independently of *Hox* gene activity. Activation of the *Hoxa2* gene in *Hox*-negative CNC leads to major craniofacial and cerebral defects: these reproduce the malformations generated by the ablation of CNC, i.e., the absence of a face and an extended anencephaly [70,73].

Along this line, ectopic expression of *Hoxa2* in the FNC was used to unmask the molecular mechanisms by which CNC coordinates facial and brain development. These data demonstrated that in the pharyngeal region, *Hoxa2* directly targets *Sine oculis* homeobox (*Six*)*2* expression and represses its activity in mesenchyme destined to form the structures in BA2 [109].

In the head, forced expression of *Hoxa2* in the facial CNC leads to a reduction in the expression of the *Six1*, *Six2*, and *Six4* genes. These genes have a similar expression pattern in premigratory CNC cells and those colonizing the nasofrontal bud and the maxillomandibular complex. However, selective inactivation of their expression by silencing generated various deficits, ranging from a global reduction in facial skeleton size to partial truncations. Under these conditions, brain alterations in the choroid plexus agenesis, moderate defects of the septal region, and alobar holoprosencephaly, are also observed. In contrast, simultaneous inactivation of these three genes reproduced the malformations caused by *Hoxa2* and led to anencephaly. In addition, it turned out that both ectopic *Hoxa2* gene expression and inactivation of *Six* genes expanded Bmp signaling. Under these conditions, overexpression of Bmp antagonists, *Noggin Gremlin* and *Dan*, was sufficient to counteract the deleterious effects of ectopic *Hoxa2* expression on brain morphogenesis. These experiments revealed that the *Six* gene family, including *Six1*, *Six2*, and *Six4,* cooperate to achieve normal brain development. The identification and detailed analysis of the impact of this molecular cascade on brain morphogenesis documents the mechanisms mobilized by the CNC to promote the sophistication of anterior brain vesicles [110]. These results suggest that during evolution, the diversification of *Six* gene function in *Hox*-negative CNC cells has enabled the elaboration and development of complex craniofacial and cerebral structures.

## 12. Integration of Fgf, Wnt and Bmp Signaling by the CNC for Forebrain Development

Surface Plasmon Resonance analysis revealed that Fgf8 is a high-affinity ligand for Cubullin (Cubn) and that Fgf8–Cubn binding is required for Fgf pathway transduction by the signaling mediators *Mitogen-activated protein kinase* (*Mapk*) and *Mothers against decapentaplegic homolog 1* (*Smad1*). Gene expression pattern shows that *FgfR1*, *FgfR3*, and, to a lesser extent, *FgfR2,* are expressed in CNC cells to a pattern similar to Cubn. Silencing of *FgfR3* at the neurulation stage results in microcephaly and facial hypoplasia, which is as severe as the effects following Cubn inactivation, whereas inactivation of *FgfR1* or *FgfR2* does not affect head development. Simultaneous inactivation of *Cubn* and *FgfR3* worsens the microcephaly phenotype: the addition of the deficits indicates that Cubn and FgfR3 are partners in Fgf8 signaling at these early stages. Together, they are required for integration of Fgf8 signals into CNC cells to orchestrate face and brain development [92,95,111]. These experiments showed how the highly conserved function of this receptor maintains the survival of the anterior neural plate and allows specification of the prosencephalon.

To further highlight the role of CNC on forebrain development, we analyzed whether CNC cells control the expression of transcription factor *Foxg1*, the earliest telencephalic marker [107,112]. *Foxg1* activity is known to regulate neuronal maturation: mutations in this gene in humans are associated with microcephaly and mental retardation [108,113]. Previous work demonstrated that *Foxg1* expression in the telencephalon is linked to Fgf8 activity in the ANR [114,115,116,117]. To explore the link between CNC and *Foxg1* expression, the activity of the *Smad1* was modulated in FNC. Smad1 is an intracellular transduction factor for Fgf, Bmp, and Wnt signaling, which is transiently expressed by CNC cells just before their migration. To unmask the mechanisms by which CNC exerts its trophic role on the telencephalon, *Smad1* activation was blocked: CNC cells became insensitive to Bmp, Fgf, and Wnt signaling and stopped their morphogenetic activity on the brain.

**Figure 6:**
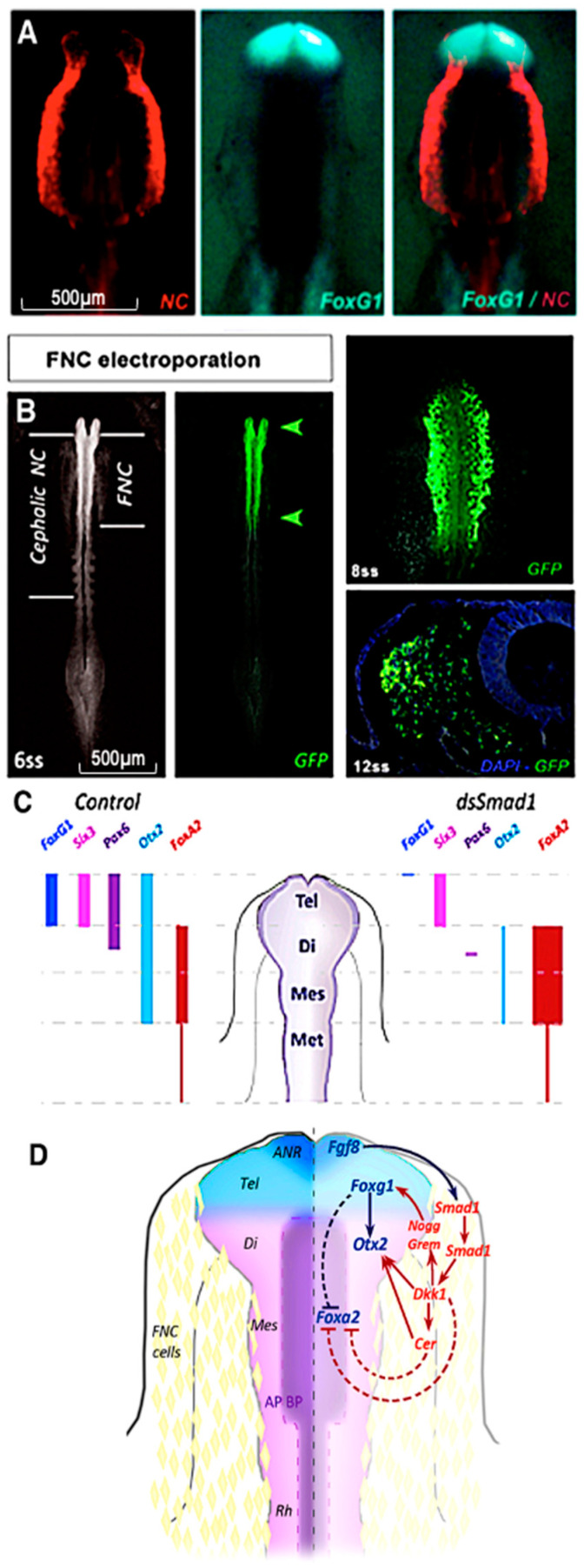
FNC controls *Foxg1* expression. (**A**) NC cell migration (red) coincides with *Foxg1* activation (cyan). (**B**) RNAi electrophoresis against *Smad1* in the NC inhibits (**C**) expression of *Foxg1*, *Otx2*, and *Pax6*. (**D**) Regulation of *Foxg1* by FNC mobilizing *Dkk1* to control *Gremlin* and *Noggin* activity, and of *Otx2* by *Cerberus* that enhances *Foxg1* expression. Adapted from [120].

Inactivation of *Smad1* led to the loss of *Fgf8* and *Foxg1* expression. This resulted in severe microcephaly and partial holoprosencephaly observed from E4 to E8. Transfection of CNC cells with constructs that drive expression of constitutively active forms of *Smad1*, mimicking the activation of the Fgf and Wnt pathways [118], recovers *Foxg1* expression and rescues telencephalon development. Furthermore, the loss of *Foxg1* function in the telencephalon affected the development of the thalamus and optic roof by deregulating gene expression at their level: *Orthodenticle homeobox* (*Otx*)*2* was reduced dorsally, and conversely, *Foxa2* significantly increased in the basal plate, meaning that this part of the brain is ventralized in the absence of telencephalic development (Figure 6). In addition, downstream of *Smad1* activity in CNC cells, *Dickkopf-related protein* (Dkk1), an antagonist of Wnt signals, was shown to control the induction of telencephalon specification through *Foxg1* activation [119]. Moreover, *Cerberus*, an antagonist of the Bmp pathway, was also required, and acted synergistically with *Dkk1* to control *Foxg1* expression, maintain *Otx2* expression, and repress the ventral expansion of *Foxa2* ([119]; Figure 6).

## 13. Concluding Remarks and Perspectives

Developmental studies in embryos point to the CNC cells’ role in brain growth and specification and highlight the molecular mechanisms by which CNC exerts a “paracrine” pro-encephalic control on brain regionalization. Altogether, these data show that the FNC plays a crucial role in controlling *Foxg1*, *Otx2*, *Emx2*, and *Dlx2* genes’ expression, which is essential for early brain development [99,120]. They shed new light on the molecular control of these transcription factors crucial for forebrain development and the development of cognitive function at birth.

In humans, mutations in *Foxg1* and 14q12 microdeletion are responsible for microcephaly, mental retardation, language and manual skills’ regression [108,113]. Epidemiologic studies in humans have highlighted that several neurodevelopmental syndromes or disorders have neurocristopathy manifestations before cognitive impairment at birth. For instance, *Foxg1* mutations are responsible for microcephaly and mild facial dysmorphisms in the atypical form of Rett syndrome and 14q12 microdeletion [108]. Septo-Optic Dysplasia [121], caused by mutations in *Otx2*, triggers syndromic phenotypes, including midline defects. These include agenesis of the interhemispheric septum and/or corpus callosum and lead to cortical malformations, microphthalmia or anophthalmia, and intellectual deficits [122,123]. Mutations in the homeobox gene *Emx2* led to severe schizencephaly in humans, characterized by full-thickness cleft within the cerebral hemispheres [120]. Additionally, both *Pax6* and *Dlx2* have been linked to the pathogenesis of autism spectrum disorders. Some studies emphasized the role of the *Pax6* gene as a chromatin modulator of autism-related genes [124], and *Dlx2* is associated with an increased susceptibility to autism [125]. Overall, studies by Creuzet and coworkers showed that FNC plays a crucial role in controlling *Foxg1*, *Otx2*, *Emx2*, and *Dlx2* genes’ expression, which is crucial for early brain development [99,120], suggesting a possible role of CNC dysfunctions in the etiology of some neurodevelopmental defects and encephalopathies.

## Data Availability

Not applicable.
